# Efficacy of a Supervised Exercise Program on Pain, Physical Function, and Quality of Life in Patients With Breast Cancer: Protocol for a Randomized Clinical Trial

**DOI:** 10.2196/63891

**Published:** 2025-03-12

**Authors:** Jennifer García-Molina, Olalla Saiz-Vázquez, Montserrat Santamaría-Vázquez, Juan Hilario Ortiz-Huerta

**Affiliations:** 1 Paseo de los Encomendadores Faculty of Health Sciences University of Burgos Burgos Spain

**Keywords:** breast cancer, exercise, quality of life, muscle strength, pain, efficacy, protocol, physical exercise, fatigue, loss of muscle, physical function, randomized clinical trial, patients with cancer

## Abstract

**Background:**

Breast cancer is the second most common cancer in women worldwide. Treatments for this disease often result in side effects such as pain, fatigue, loss of muscle mass, and reduced quality of life. Physical exercise has been shown to effectively mitigate these side effects and improve the quality of life in patients with breast cancer.

**Objective:**

This randomized clinical trial aims to evaluate the efficacy of a 12-week supervised exercise program on pain, physical function, and quality of life in female patients with cancer.

**Methods:**

This randomized, double-blind clinical trial will recruit 325 participants, divided into an intervention group receiving the exercise program and a control group receiving standard care recommendations. Outcome measures, including pain (assessed via the Brief Pain Inventory), physical function (Disability of the Arm, Shoulder, and Hand Questionnaire), and quality of life (European Organization for Research and Treatment of Cancer QLQ-C30 and European Organization for Research and Treatment of Cancer QLQ-BR23), will be evaluated at baseline, immediately post intervention, and 12 weeks post intervention. Statistical analysis will involve repeated measures of ANOVA and MANOVA to determine the significance of the intervention’s effects across time points.

**Results:**

Recruitment and data collection will commence in February of 2025, and data analysis is scheduled for completion at the end of 2025. No results are currently available

**Conclusions:**

Physical exercise is anticipated to play a significant role in alleviating pain, enhancing physical function, and improving the quality of life in female patients with cancer. This study will provide robust evidence to support the integration of supervised exercise into standard care protocols for this population.

**Trial Registration:**

ClinicalTrials.gov NCT06618690; https://clinicaltrials.gov/ct2/show/NCT06618690

**International Registered Report Identifier (IRRID):**

PRR1-10.2196/63891

## Introduction

### Background

The World Health Organization (WHO) defines breast cancer as a pathology in which cells present in the breast reproduce uncontrollably and form tumors [1]. It is the second most common type of cancer worldwide, with an incidence in 2022 of 2.3 million. Similarly, in 2022, its mortality rate ranked fourth in the world with a rate of 669,418 [2]. Women are the most affected by this disease, with only 0.5% to 1% of cases in men [1].

Genetic mutations play a key role in cancer development. Mutations in *BRCA1* and *BRCA2* increase the risk of breast [3], ovarian, and prostate cancers, while *TP53* is linked to multiple cancers like breast and colon due to its role in cell cycle regulation [4]. *PTEN* mutations are found in breast and thyroid cancers [5], and APC is associated with colorectal cancer [6]. *MLH1* and *MSH2* mutations cause Lynch syndrome, increasing colorectal cancer risk [7]. *KRAS* mutations are common in lung, colon, and pancreatic cancers [8].

Other causes of the disease are diverse: on the one hand, advanced age at motherhood and late menopause are also considered risk factors. On the other hand, breastfeeding, physical exercise and not consuming alcohol are considered protective factors against the disease [9,10].

Treatment will be selected according to the characteristics of the tumor. Generally, it is based on neoadjuvant or adjuvant chemotherapy, radiotherapy, surgery, and hormonal therapy [11].

The prognosis of the disease has improved due to early diagnosis and therapeutic advances. The mortality rate, in turn, has decreased significantly [12,13]. Because of this increase in survival, scientific society is becoming interested in the treatment of the side effects that these patients may suffer, which include both physical and psychological symptoms [14]. Associated physical symptoms that may appear include lymphedema, decreased movement and strength of the upper limb, decreased mobility and strength of the upper limb [15], pain [16], bone loss [17], and sarcopenia [18].

These side effects have a direct impact on the health-related quality of life of patients with cancer [14]. The WHO defines quality of life as the individual’s perception of his or her social and cultural way of life, as well as his or her expectations and goals. This is a very open concept that encompasses different aspects of the person from physical health, psychological state, level of independence, social relationships, and personal beliefs [19]. Quality of life measurement is frequently used in breast cancer studies to evaluate the effectiveness and outcomes of treatments [20].

### Exercise and Breast Cancer

It has been observed that patients with breast cancer present atrophy of the skeletal muscle apparatus, which is associated with the mitotoxic effects of chemotherapy. Anthracyclines, substances that play a leading role in chemotherapy against breast cancer, have an affinity for the inner membrane of the mitochondria, the place where the body’s energy is produced; therefore, this mechanism explains fatigue, atrophy, and muscle pain [21,22].

Various treatments are currently being studied to alleviate these side effects, including therapeutic exercise. Exercise provides short- and long-term benefits to reduce the symptoms that may appear during treatment in each patient. Here, therapeutic exercise specialists play an important role in helping patients overcome their fear and improve their physical abilities [23]. The American College of Sports Medicine says exercise is associated with improved survival after developing cancer [24].

The health professionals in charge of applying this type of therapy must evaluate each patient individually and adapt the exercise prescription to each one of them. The ideal type of exercise for these patients is one that includes a combination of aerobic and resistance exercise, the intensity of each of these parts will be determined by the characteristics of the patient [25,26].

There is evidence that professionally supervised physical exercise has positive results compared to unsupervised exercise. Research has shown evidence that it positively influences decreases anxiety, depressive symptoms, and fatigue, improves quality of life and physical function, and there is no risk of exacerbating upper limb lymphedema. Even people who have already developed lymphedema can perform resistance exercises. In 2000, Harris and Niesen-Vertommen [27] were the first to start strength studies with people with lymphedema, as the existing recommendations so far were the rest of the affected limb [27-30].

The objective of this project is to develop a randomized clinical trial to determine the efficacy of a supervised exercise program on pain, physical function, and quality of life in female patients with cancer.

## Methods

### Hypothesis

- A supervised exercise program reduces pain and improves physical function and quality of life in female patients with cancer compared to a control group not receiving the exercise program.
- The beneficial effects of the supervised exercise program are maintained over time at least 12 weeks after the end of the program.

### Study Design

**Figure 1:**
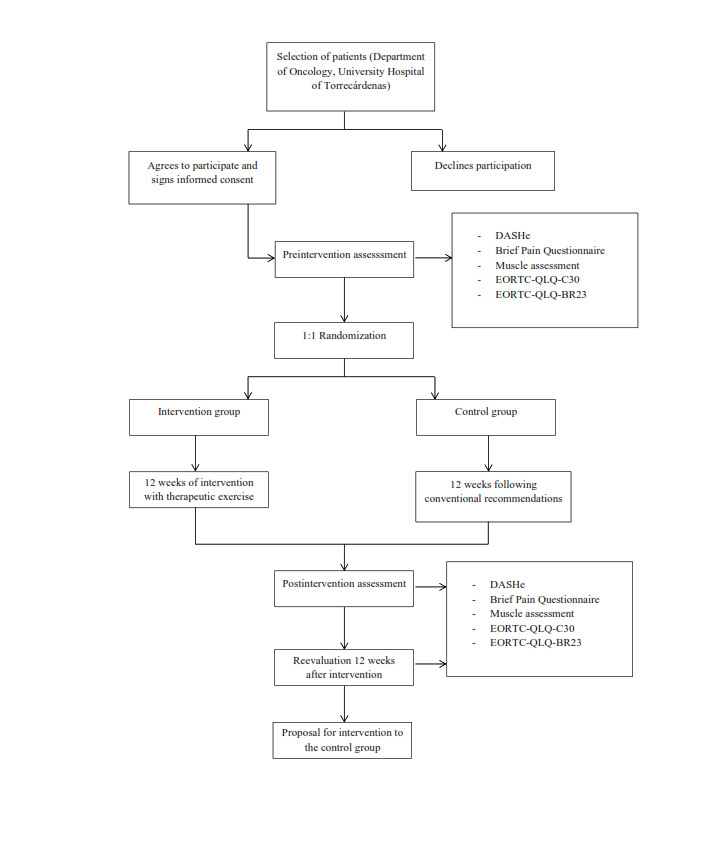
Untitled.

This study will be a single-blind, randomized clinical trial. To determine the efficacy of a therapeutic exercise program on the different dimensions of quality of life, physical function, and pain in patients with cancer. Once the participants have been recruited, they will be randomly assigned to 2 groups (intervention group and control group). Three assessments will be made: initial assessment, assessment after the end of the intervention, and assessment 12 weeks after the end of treatment. The intervention group will follow a 12-week therapeutic exercise plan, and the control group will follow the usual recommendations for this type of patient. After this last assessment, the participants in the control group will be offered the intervention. (Figure 1). Approval will be sought from the ethics committee of the University Hospital of Torrecárdenas.

### Study Population

The participants will be recruited through the oncologists of the University Hospital of Torrecárdenas. The inclusion criteria will be the following: women aged between 45 and 65 years (age range with the highest incidence in Spain), with oncologic treatment completed less than 3 months ago, without metastasis, without pathologies that contraindicate exercise, and who have agreed to sign the informed consent form. The exclusion criteria will include: not attending scheduled sessions to instruct on the exercises to be performed, being part of other studies, undergoing another type of therapy, and pregnant patients.

The selected age range of 45 to 65 years for participants is based on epidemiological data indicating that this demographic encompasses the highest incidence of breast cancer in Spain. This range ensures a representative sample for evaluating the intervention's efficacy while excluding populations less likely to face similar diseases and treatment dynamics. Additionally, menopause is included as an independent variable in the analysis due to its significant influence on both physiological and psychological responses to breast cancer treatment and exercise interventions. Hormonal changes associated with menopause can affect musculoskeletal health, fatigue levels, and overall quality of life, making it a critical factor to consider when evaluating the intervention’s outcomes on pain, physical function, and quality of life [31]. By accounting for these variables, the study aims to provide a nuanced understanding of how menopausal status may modulate the benefits of supervised exercise in patients with cancer.

### Sample

To calculate the sample size, the QuestionPro software (QuestionPro Inc) was used [32]. It consists of software that, among other functions, incorporates a sample calculator for research.

Based on breast cancer morbidity data in the province of Almeria for 2022, published by the National Institute of Statistics [33], the calculation assumed a moderate effect size of 0.5, based on prior research on exercise interventions in similar populations. To ensure sufficient sensitivity to detect meaningful differences, the significance level (α) was set at .05 (2-tailed), and the study power (1–β) was set at 80%. Considering these assumptions, an initial sample size of 280 participants was determined. However, recognizing the potential for participant dropout, which is common in exercise-based trials, we accounted for an estimated attrition rate of 15%. To compensate for this and maintain statistical power, the final sample size was adjusted to 325 participants.

This approach ensures that the study is adequately powered to detect significant differences between the intervention and control groups for the primary outcomes. If further specifics or expanded parameters are required, we are happy to provide additional details. This explanation aims to address editorial concerns and clarify the robustness of our calculation methodology.

In the beginning, an attempt will be made to recruit the estimated size of participants; if this cannot be done, a smaller sample size will be used, assuming a decrease in the confidence level of the results.

### Randomization

All participants have the same probability of belonging to either group. Assignments will be randomized with a 1:1 ratio using the OxMaR software (OxMaR Inc) in its Spanish version [34].

### Measuring Tools for Evaluation

#### Disability of the Arm, Shoulder, and Hand Questionnaire

Self-administered and validated questionnaire in Spanish, the original questionnaire was first published in 1996 [35]. This measuring instrument assesses the impact of processes occurring in the upper limb. This questionnaire consists of 30 items and 2 optional modules. The score obtained is transformed to a scale from 0 to 100, where higher scores show a worse result. It shows a high internal consistency (Cronbach α=0.96) [36].

#### Brief Pain Questionnaire

This questionnaire is a pain assessment instrument used to measure pain intensity and its impact on the patient’s daily life; the original questionnaire was developed at McGill University in 1971, with an internal consistency of 0.7 [37]. The questionnaire used in the study was validated in Spanish in patients with neoplastic pain. It consists of 11 items on a Likert-type scale from 0 to 10 (where 0=no pain and 10=worst possible pain) and 15 additional items that assess the impact of treatment on pain relief. It presents a high internal consistency, with Cronbach α being higher than 0.7 in all dimensions (0.89 for pain intensity and 0.87 for impact on daily life) [38].

#### Muscle Assessment

Muscle strength will be assessed using a 1 maximum repetition (MR) protocol for the chest press in the horizontal plane and the leg press, as this is the most commonly used muscle assessment test in similar studies, according to the meta-analysis by Hasenoehrl et al [39]. One RM is defined as the maximum weight at which only 1 repetition can be performed [40].

For the evaluation, we will start with an initial warm-up series with 6 repetitions, the initial weight will be 2.5 kg and 18 kg for the upper and lower limbs, respectively. According to the perceived effort of each participant, we will start with an approximate weight for the one RM test, until it increases according to tolerance to find out the 1 RM weight of each participant. This is the strategy followed by Brown and Schmitz [41]. The assessment of the upper limb will be performed unilaterally in order to objectively evaluate the difference between the affected and healthy upper limb, as was done by Hagstrom et al [42]. Thus, we will start with 1.25 kg for each upper limb.

#### EORTC-QLQ-C30 Quality of Life Questionnaire

A questionnaire was created by the European Organization for Research and Treatment of Cancer (EORTC) to assess the quality of life in patients with cancer. The organization provides the questionnaire translated into several languages, including Spanish. It consists of 30 Likert-type questions that assess various aspects of quality of life, including the ability to perform activities, symptom control, general perception of health, and other relevant aspects such as difficulty breathing, insomnia, anorexia, constipation, diarrhea, and economic impact [43].

The EORTC-QLQ-C30 presents in most of the studies performed a good internal consistency (Cronbach α=0.7 for most of the scales). This questionnaire, based on a specific formula, obtains results on a scale from 0 to 100, where a higher score indicates a better quality of life. The scores for each question within each dimension (physical, emotional, and social functioning) are summed [44]. Within this questionnaire, there are specific modules that complement it for some types of cancer, for breast cancer they have created the EORTC QLQ-BR23 [44].

#### EORTC Quality of Life Questionnaire, QLQ-BR23

This questionnaire is a specific module for breast cancer that complements the previous questionnaire. Two main aspects are assessed here: functional scale (body image, sexuality, and concern for the future) and symptom scale (breast symptoms, arm, and side effects of treatment). It presents in most of the studies carried out a good internal consistency (Cronbach α=0.7) [44]. Like the general module, this questionnaire has a score from 0 to 100, where they are transformed according to a formula by specific dimensions.

### Ethical Considerations

In this study, clinical investigations involving medical devices are conducted on human participants. In order to safeguard the integrity, well-being, and privacy rights of the participants, the study is carried out in accordance with the guidelines set forth by the Declaration of Helsinki of the World Medical Association (64th General Assembly, Fortaleza, Brazil, October 2013), the Council of Europe Convention on Human Rights and Biomedicine, Good Clinical Practice standards, Royal Decree 1090/2015 of December 4, which regulates clinical trials with medicinal products, the Research Ethics Committees for Medicinal Products and the Spanish Registry of Clinical Studies, and Royal Decree 1591/2009 of October 16th, which regulates medical devices [45,46].

The study protocol will be submitted to the Bioethics Committee at the University Hospital of Torrecárdenas for review and approval in February 2025, during the preparatory phase. Ethical approval will be obtained before the initiation of any participant recruitment or data collection activities, in compliance with ethical standards. Recruitment is scheduled to begin in February 2025, contingent on receiving ethical approval. The ethical approval number will be provided in the final manuscript once it is obtained.

Before the study commences, explicit informed consent will be requested from participants via a digital form, clearly explaining the study’s objectives and participation details (Multimedia Appendix 1). Participants will have the ability to opt out at any stage without consequences. For any secondary data analyses, it will be confirmed that prior institutional review board–approved consent covers reuse without requiring additional consent. Compliance with Spanish Organic Law 3/2018 of December 5, 2018, on the Protection of Personal Data and the guarantee of digital rights [47], will also be ensured. Participant privacy will be rigorously protected, and all collected data will be anonymized or deidentified to prevent the disclosure of personal information. The data will be securely stored and used exclusively for this research project and any resulting publications. If any identifiable data is included in images or supplementary materials, explicit consent will be obtained and the necessary documentation will be submitted as supplementary files.

Participants will not receive any financial compensation for their participation in the study. However, they will be provided with detailed information about the study’s objectives, potential benefits, and their rights as participants to ensure transparency and fairness in the research process. These measures ensure that the study adheres to the highest ethical standards and prioritizes participant safety, confidentiality, and informed consent in compliance with national and international research ethical regulations.

### Intervention

The proposal will be carried out in the intervention group (group 1), while the control group (group 2) will undergo the conventional recommendations indicated for this type of patient.

A supervised training plan will be carried out for 12 weeks with a frequency of 3 sessions per week with a duration of 60 minutes, an ideal estimate according to the meta-analysis of Montaño-Rojas et al [48]. In first 2 weeks, the group exercises will be performed to familiarize the participants with the exercises and then each participant will perform them at home with a review every 2 weeks to assess the increase of the load.

The exercises and loads included in this treatment plan have been designed and modified based on previous clinical trials [39,49-51]. The training plan will work the upper limbs, lower limbs, and trunk. All sessions begin with a general warm-up, which will be previously instructed to the participants, in which the heart rate will be increased (Multimedia Appendix 2). After a 2-minute break, the specific block of each session will begin (Textbox 1 and Multimedia Appendix 2). All exercises will be performed with dumbbells, elastic bands, and a ball. We will consider the age of each participant, making minor adjustments to the initial exercise program as needed.

Three sets of all exercises will be performed with 10-12 repetitions at 50%-60% 1 MR (previously calculated for each participant in each of the exercises) with 1 minute of rest between sets. Finally, we will conclude with general stretching previously instructed (Multimedia Appendix 2). The control group will participate in a general education and wellness program designed to promote overall well-being and maintain a basic level of activity. This program, matched in duration to the experimental group, will include regular counseling sessions covering topics such as nutrition, stress management, sleep quality, and relapse prevention. In addition, participants will be encouraged to engage in low-impact physical activities, such as short walks and gentle mobility exercises. For the experimental group, the supervised exercise program aims to improve pain, physical function, and quality of life through a variety of carefully selected exercises tailored to each participant’s needs. These include stretching exercises, such as pectoral stretches for chest mobility and shoulder and arm stretch to enhance the range of motion and relieve tension. Strength training exercises using resistance bands or light weights will focus on improving upper limb and core strength to enhance posture and stability. Aerobic activities like walking or stationary cycling will be incorporated to boost cardiovascular endurance and mobility. Flexibility exercises, including range-of-motion activities, will help maintain and improve overall flexibility, while balance and coordination exercises will enhance stability and prevent falls. Muscle endurance will also be developed through lightweight or resistance band exercises. The program's design emphasizes a personalized approach, guided by physiotherapists or exercise specialists, to ensure safety and maximize benefits for patients with cancer.

**Strength exercises included in the training plan.**

**Session 1**

- Squats
- Biceps curl
- Superman in quadruped
- Dumbbell chest press in horizontal plane
- Shoulder rotators with elastic band
- Dumbbell rowing

**Session 2**

- Proprioceptive shoulder exercises with ball on wall
- Static stride
- Upper limb flexion with ball on wall
- Isometric abduction with ball (elbow 90º)
- Prone spine extension
- Quadruped hip abduction

**Session 3**

- Dumbbell triceps extension
- Quadruped gluteal kick
- Triceps kick with dumbbell
- Lateral shoulder raises with dumbbells
- Dead weight with dumbbells
- Rowing with elastic band

### Data Management and Analysis

Once all the measurements included in the measuring instruments section have been taken, they will be recorded and analyzed using SPSS (version 28.0; IBM Corp) [52].

The quantitative data of the sample will be described by number, mean, and SD.

A result is understood to be significant when *P*≤.05. In statistics, significance or internal consistency indicates that there is congruence between the different results obtained [53].

For the statistical analysis of the results of this test, 2 tests will be performed as follows.

- Repeated measures ANOVA: This analysis allows us to evaluate whether the differences between the results of the same test in a participant are statistically significant. If so, it also determines what percentage of the effect is attributable to the intervention and not to other factors [53]. Two ANOVA analyses will be performed: one comparing the preintervention assessment with the postintervention assessment, and the other comparing the postintervention with the assessment 12 weeks later. This test will check if there is a significant improvement in the different measures of pain, physical function, and quality of life. Finally, the paired comparison table will tell us in which measurement periods the differences are found [53].
- ANOVA of differences: In this case, the differences between the results of the intervention group and the control group will be compared [53]. With the results obtained from this statistical analysis, it will be possible to verify whether the differences between groups in terms of pain, physical function, and quality of life are significant between the participants receiving the intervention and those belonging to the control group.
- MANOVA: To assess the overall effect of the intervention and determine whether the hypotheses can be accepted or refuted, the following hypotheses will be tested [53].

These statistical analyses described the significance between the test results of the same individual and the significance between the different results of both groups of the test that will be tested.

## Results

Recruitment and data collection will begin in the third quarter of 2024. Data analysis will be conducted between the first and second quarters of 2025. No results are available at the time of this intervention protocol.

## Discussion

### Principal Findings

This randomized clinical trial is intended to test the efficacy of an exercise program applied to patients with cancer.

Most of the patients present a deterioration of their quality of life and physical function after suffering breast cancer, physical exercise can be a great ally to counteract these negative effects [23-30]. Therefore, the results obtained in the intervention group are expected to be significantly better than those of the control group. It is likely that there will be benefits to the intervention in some aspects and not in all the variables measured in the study. In addition, the benefits provided are expected to be maintained in the long term, hence the reassessment 12 weeks after the end of the intervention.

A compelling finding emerges from the systematic review by Montaño-Rojas et al [48], which demonstrates significant muscle strengthening benefits, even with low-intensity and short-duration exercise programs. Participants in these programs showed notable improvements in muscle strength compared to those who did not engage in physical activity. This aligns with the meta-analysis by Shen et al [54], which reported improvements in pain and joint amplitude of the upper limb following physical exercise after radiotherapy in breast cancer. These findings complement international guidelines, such as those from the Clinical Oncology Society of Australia (COSA), which recommend at least 150 minutes of moderate aerobic exercise and 2 resistance training sessions per week, tailored to the individual’s health and treatment phase. Like the protocol in this study, COSA highlights the benefits of exercise in reducing fatigue, improving musculoskeletal health, and enhancing quality of life [30]. However, COSA also emphasizes the routine integration of exercise into oncology care, a practice that remains inconsistently implemented across European health care systems. This comparison underscores the universal importance of exercise in patients with cancer and highlights the need for tailored adaptations to local health care systems and cultural contexts to maximize accessibility and impact [29].

In line with the aforementioned, Zengin Alpozgen et al [55] obtained positive results in their clinical trials for pain relief. In contrast, the results of Loudon et al [56] are not significant in terms of pain reduction. This difference may be due to the fact that the first two use exercises that involve greater resistance and dosage; in contrast, the latter performs an intervention of a single session per week based exclusively on yoga exercises.

On the one hand, Aydin et al [57] in their clinical trial, show that physical exercise not only has a positive influence on the physical condition of the participants but is also strongly related to the improvement of mental health. In her study with 48 participants, she used the EORTC-QLQ-C30 and WHO Quality of Life–Brief Version scales to measure the quality of life of the sample. On the other hand, Koevoets et al [58] used the EORTC-QLQ-C30 scale to measure the impact of exercise on cognitive function in patients with breast cancer after chemotherapy. Reporting positive results in the participants of the intervention group. On the contrary, Bruce et al [59] in a clinical trial with 392 participants, found no significant differences in the improvement of mental health in participants who had performed physical exercise and those who had followed the usual recommendations. These differences may be due to the sample size as the latter study presents a significantly larger sample than the previous ones. They may also be due to the dosage and type of exercises proposed in the intervention; since Aydin et al [57] and Koevoets et al [58] perform aerobic and general strength exercises of all muscle groups, while Bruce et al [59] only perform a shoulder strengthening program in flexion, abduction, and external rotation.

Regarding when to start the exercise program, authors such as Carayol et al [60] recommend starting a supervised exercise program during chemotherapy or radiotherapy treatment, combined with an adequate diet to improve health status and the consequences of these treatments.

Vincent et al [61] compared in a randomized clinical trial the results obtained in a group that started the intervention with exercise during chemotherapy and in another group that started it after the end of chemotherapy, not observing significant differences between the two and highlighting the importance of performing them either during or after.

From another point of view, not only is the impact of physical exercise after breast cancer being investigated but there is already research that is beginning to show positive results in the prevention of breast cancer through exercise in populations with risk factors. An example of this is the studies by Coletta et al [62] and Khosravi et al [63] where they performed interventions with physical exercise in postmenopausal participants and with various other risk factors to see the impact of this training with leptin and myokine levels. Prevention of both first-time disease onset and recurrence, aided by physical exercise could be a future line of research that will gain more momentum.

The strengths of this study are randomization, as well as the use of previously validated assessment tools, some of them specific to breast cancer, such as the EORTC QLQ-BR23. The variables analyzed range from pain to physical function and quality of life. In addition, it is based on a protocol of resistance exercises, which, according to previously cited research, provides better benefits.

The main limitation of this study is that the intervention will be carried out through patients recruited from a single referral hospital, although a large sample is achieved, it would be interesting for future studies to have a variety of more heterogeneous participants and better generalize the results. In addition to making longer-term measurements to see how the possible improvements acquired are maintained. Furthermore, the study’s results may be influenced by factors like hormonal variables, age, and treatment responses. Hormonal changes, such as menopause, can affect treatment effectiveness and side effects. Additionally, younger patients may experience different side effects or treatment responses due to metabolism, health, and comorbid conditions.

### Conclusions

Oncology research is advancing at a very fast pace. Therefore, addressing the side effects of the disease is a great success for these patients, who today have a high survival rate.

Therapeutic exercise is a great ally in improving strength, pain, and joint mobility after breast cancer, as well as positively influencing the improvement of quality of life.

## Appendix

Multimedia Appendix 1 Informant consent.

Multimedia Appendix 2 Exercises session.

## Abbreviations

COSA: Clinical Oncology Society of Australia
EORTC: European Organization for Research and Treatment of Cancer
MR: maximum repetition
WHO: World Health Organization

## Data Availability

The data that will support the findings of this study will be derived from patient medical record data. The datasets generated during and/or analyzed during this study are available from the corresponding author on reasonable request.
