# Supplementary material for: Efficacy of a Supervised Exercise Program on Pain, Physical Function, and Quality of Life in Patients With Breast Cancer: Protocol for a Randomized Clinical Trial
Source: JMIR Res Protoc. 2025 Mar 12;14:e63891. doi: 10.2196/63891 (PMC11947629; doi:10.2196/63891)
Supplement: Multimedia Appendix 1 [file resprot_v14i1e63891_app1.docx]

APPENDIX 1

INFORMANT CONSENT

1.- PARTICIPANT INFORMATION IN THE RESEARCH PROJECT
The research project for which we are asking for your participation is titled:
**“Efficacy of a supervised exercise program on pain, physical function, and quality of life in breast cancer survivors: protocol for a randomized clinical trial.”**

Please read the following information carefully. If you have any questions, express them to the lead investigator of the project using the contact information provided in this document before signing. If you agree with the research objectives, feel that you have received sufficient information, and wish to participate in the project, please sign at the end of this document. The consent provided here can be revoked at any time. A form to do so is included at the end of this document.

a. Study objective:

b. Methodology to be used for the study, the type of collaboration expected from you, and the duration of that collaboration:

c. Preventive, diagnostic, and/or therapeutic alternatives to the procedures being investigated in this study: (This section will be completed if applicable to the type of study being conducted).

d. Possible discomforts and risks of your participation in the study: (Indicate any health risks, especially serious ones even if infrequent; less serious risks if frequent; and personalized risks).

e. Measures to address adverse events: (In case any adverse events occur in participants, explain how they will be addressed).

f. Measures to ensure appropriate compensation in the event that you suffer any harm:

g. Expected benefits from the research:

h. Consequences of not participating: (It should be noted that choosing not to participate will not affect your right to healthcare, and your relationship with those who proposed your participation will remain as cordial and dedicated whether you decide to participate or not).

i. Possibility of withdrawal at any time and consequences: You may withdraw from the project at any time by signing the withdrawal of consent included at the end of this document. Your withdrawal will have no negative consequences for you and will be accepted without issue by the research team.

j. Who has funded the study?

k. Which institution is conducting it?

l. Compensation for participation: (Indicate whether participants will not receive any financial compensation for their participation in this study or only compensation for inconveniences).

m. Future use of the results: (Indicate whether the results will be used for educational, research, and/or scientific publication purposes).

n. Research team: (Provide the full names of the members of the research team).

o. Contact details of the lead investigator for clarifications or questions:
(Provide the full name and contact details of the lead investigator at their workplace, including phone number).

p. The project will be conducted following international ethical standards as outlined in the Declaration of Helsinki.

q. Measures to access relevant information about you that arises from the research or the overall results: You have the right to access the information generated about you in the study. If you wish to receive it, please indicate below:

☐ Yes, I wish to receive the results of the research relevant to me.
☐ No, I do not wish to receive information.
(Briefly detail how access to the research results will be provided).

r. Measures taken for anonymized studies: (Include this paragraph if it is an anonymized study. If not, delete: “An effective anonymization system has been established that does not allow the subject's identification. Under no circumstances will the consents granted, where the subject is identified, be combined with the questionnaires or other information used in the study. In the use of the study results, whether for educational, research, and/or publication purposes, personal data will remain anonymized so that the research subjects will not be identified or identifiable”).

| Name: |
| --- |
| Date: |
| City: |
| Signed: |
