# Supplementary figures and images for: Efficacy of a Supervised Exercise Program on Pain, Physical Function, and Quality of Life in Patients With Breast Cancer: Protocol for a Randomized Clinical Trial
Source: JMIR Res Protoc. 2025 Mar 12;14:e63891. doi: 10.2196/63891 (PMC11947629; doi:10.2196/63891)

APPENDIX 2


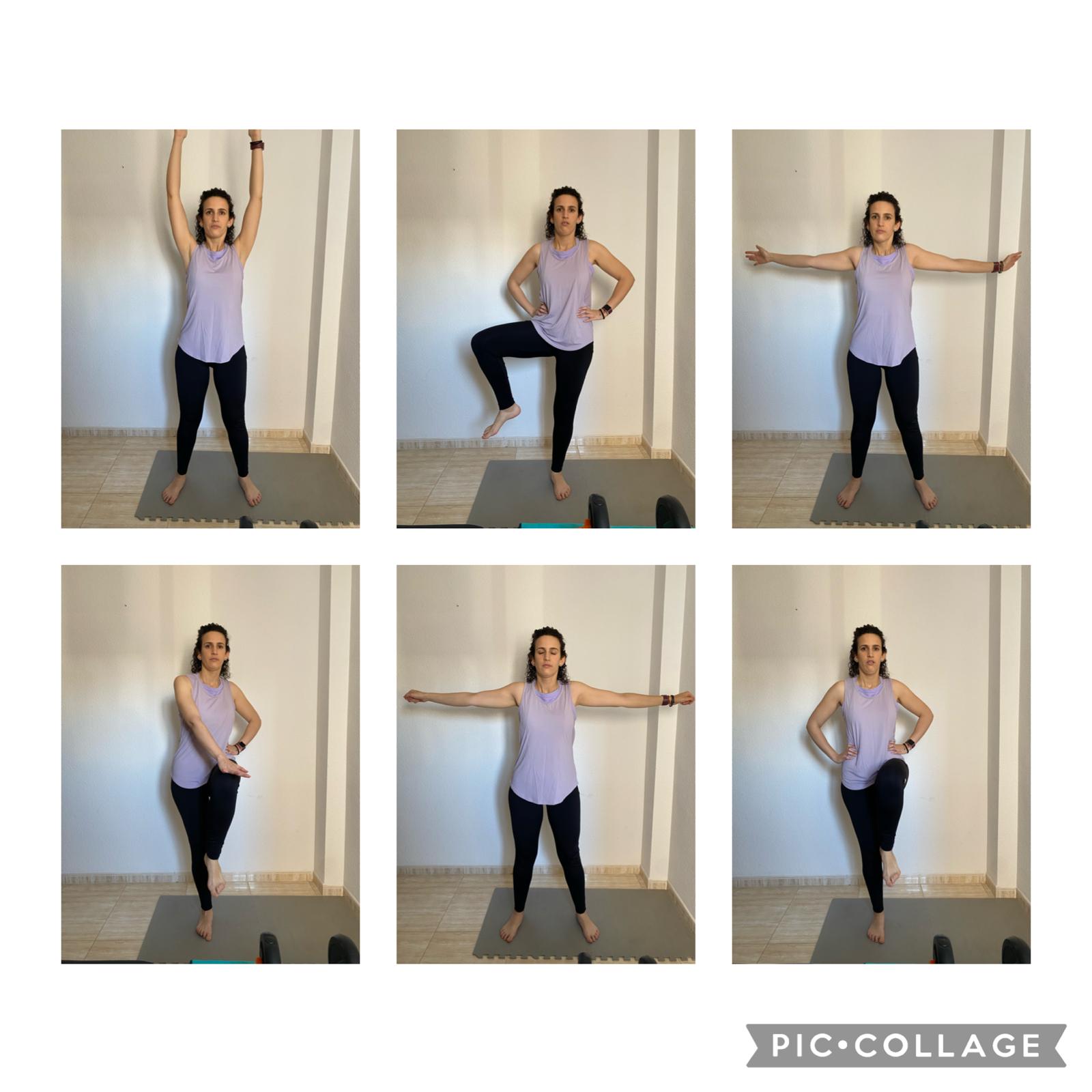
HEATING

1

2

3

5

4

6

INTERVENTION

Session 1:


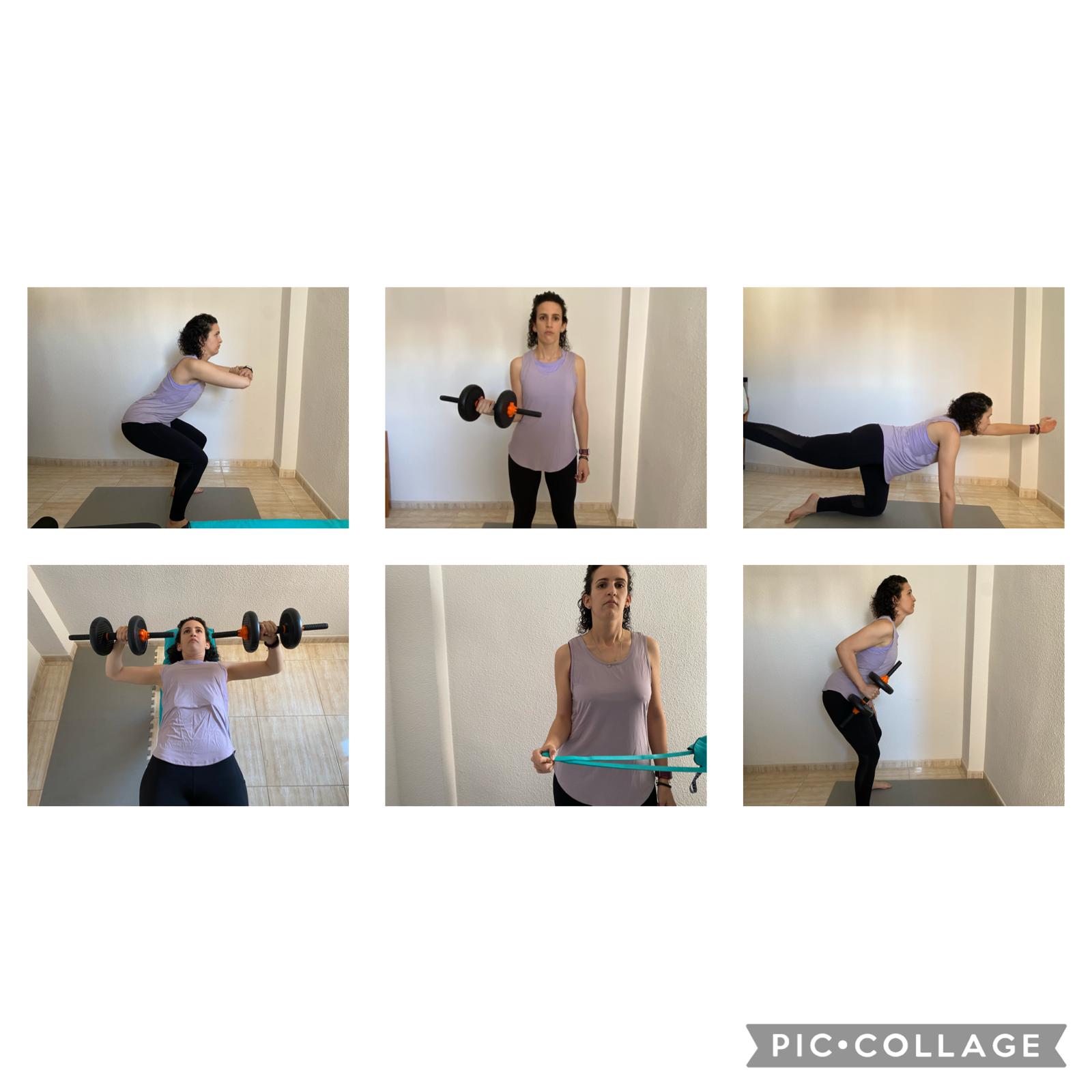


6

5

4

3

2

1

V

V

V

Session 2:


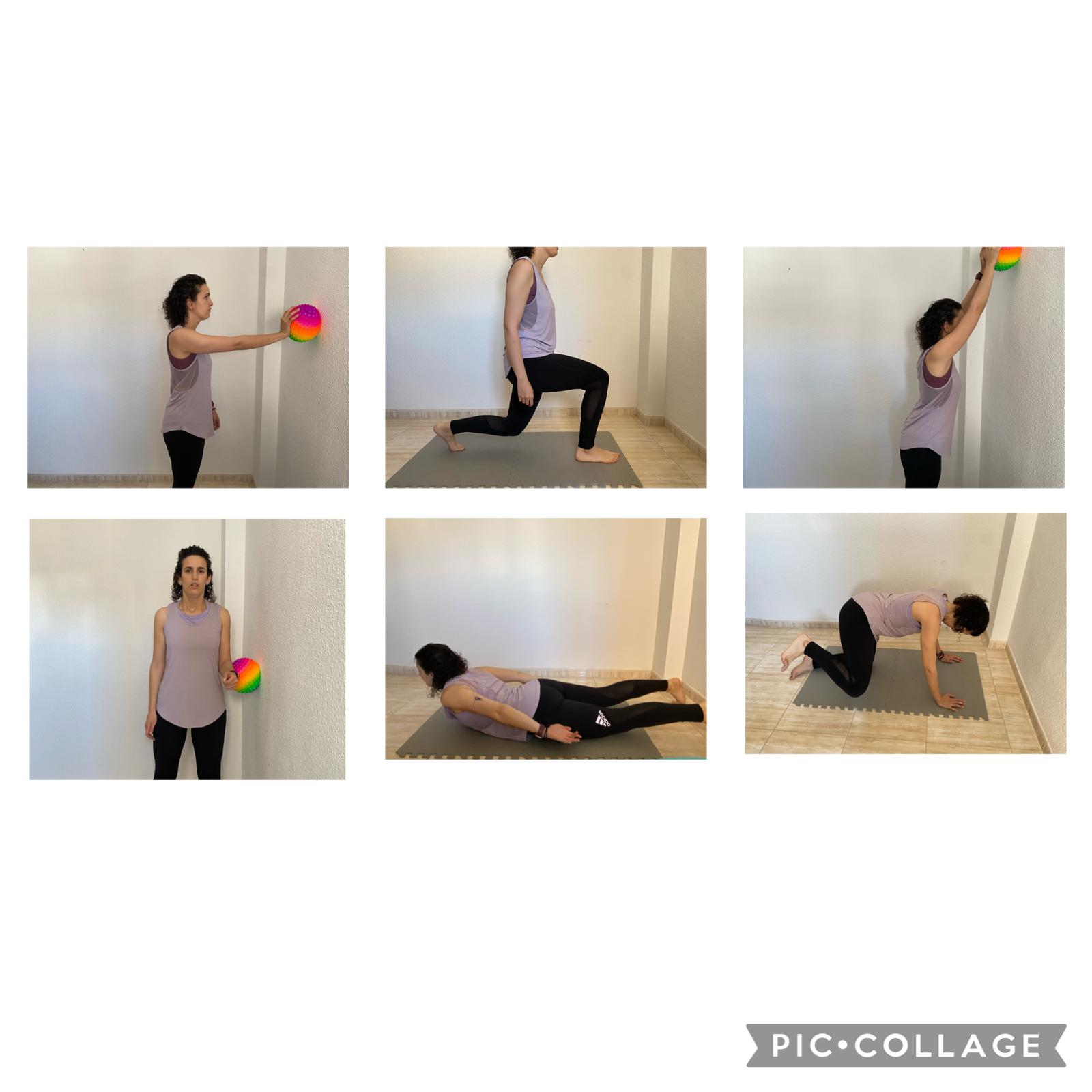


6

5

4

3

2

1

Session 3:


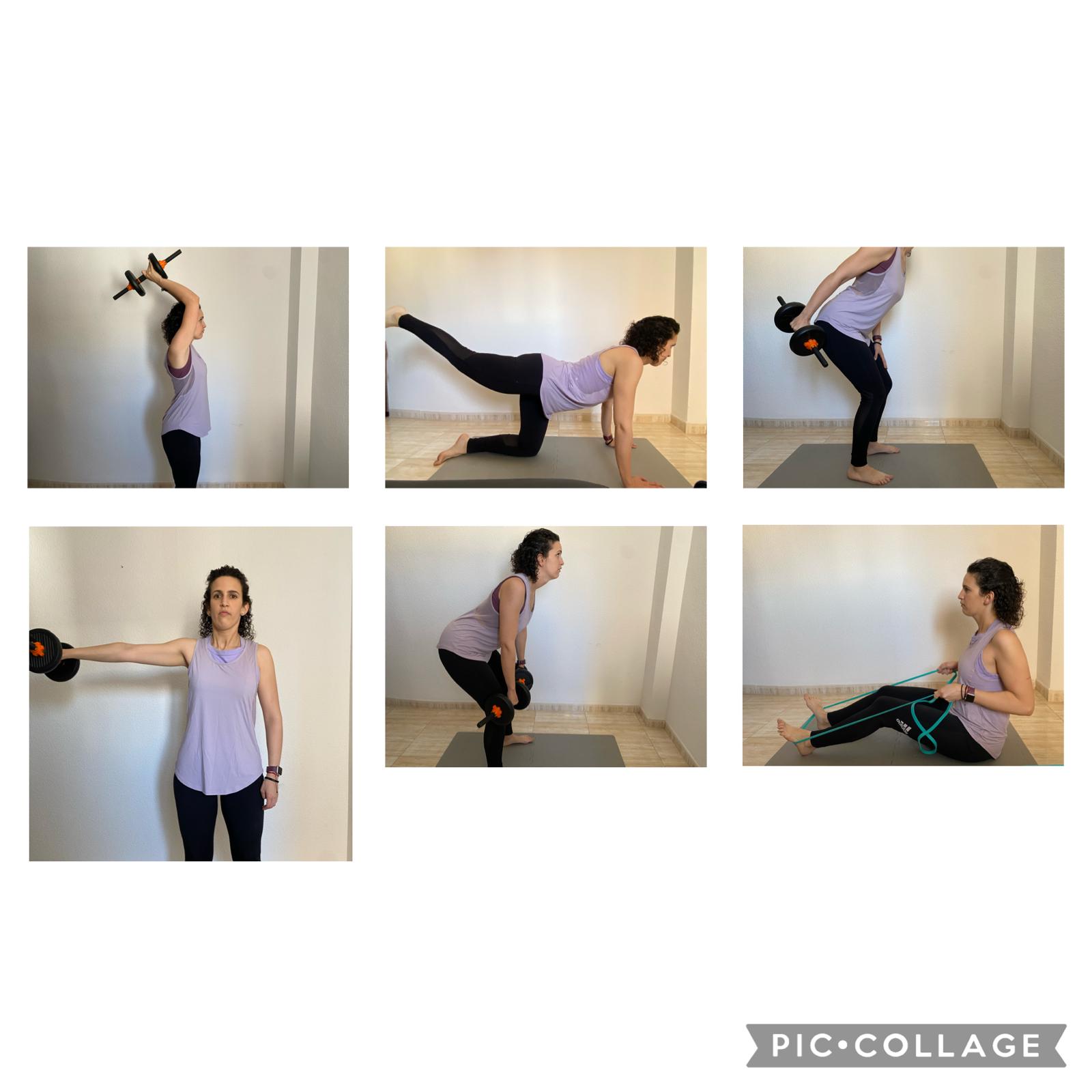


6

5

4

3

2

1

STRETCHING


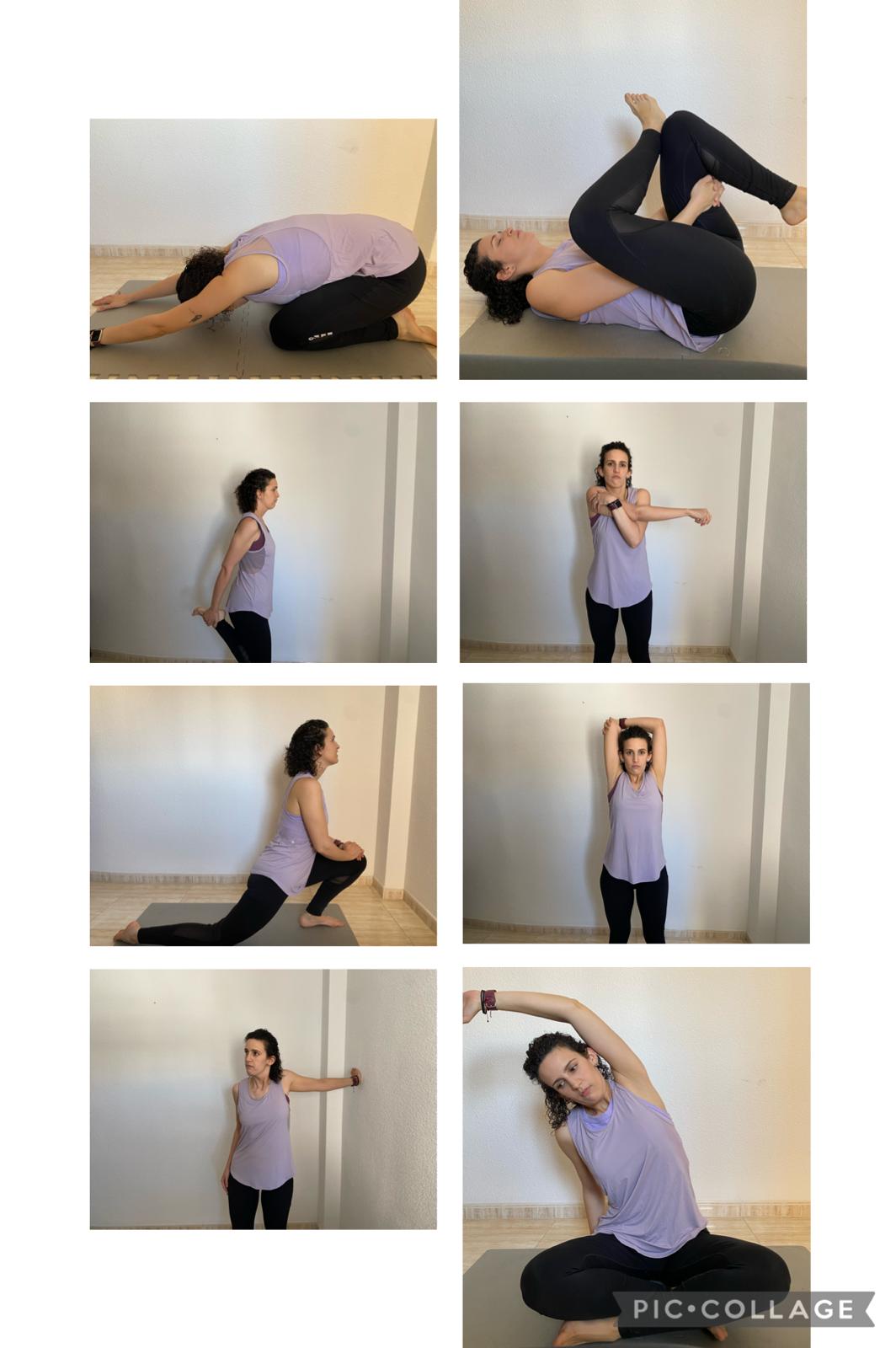


8

7

6

5

4

3

2

1

Supplement: Multimedia Appendix 2 [file resprot_v14i1e63891_app2.docx]
